# A comparison of haploidentical versus HLA-identical sibling outpatient hematopoietic cell transplantation using reduced intensity conditioning in patients with acute leukemia

**DOI:** 10.3389/fimmu.2024.1400610

**Published:** 2024-10-04

**Authors:** José Carlos Jaime-Pérez, Jorge Valdespino-Valdes, Andrés Gómez-De León, Renata Valeria Barragán-Longoria, Adriana Dominguez-Villanueva, Olga Graciela Cantú-Rodríguez, César Homero Gutiérrez-Aguirre, David Gómez-Almaguer

**Affiliations:** Hematology Department, Internal Medicine Division, Dr. José E. González University Hospital, School of Medicine, Universidad Autónoma de Nuevo León, Monterrey, Mexico

**Keywords:** hematopoietic cell transplant, outpatient transplantation, acute lymphoblastic leukemia, acute myeloblastic leukemia, reduced-intensity conditioning, peripheral blood transplant, Latin America

## Abstract

**Background:**

Hematopoietic cell transplantation (HCT) increases survival for acute leukemia. Outpatient allogeneic HCT reduces costs and increases transplant rates in developing countries. We report outcomes of outpatient HLA-identical and haploidentical HCT in acute leukemia.

**Methods:**

This single-center retrospective cohort study analyzed 121 adult patients with acute myeloblastic (AML) and acute lymphoblastic leukemia (ALL) receiving an outpatient allogeneic HCT with peripheral blood allografts after reduced-intensity conditioning (RIC) from 2012-2022.

**Results:**

There were 81 (67%) haploidentical and 40 (33%) HLA-identical transplants. Complete chimerism (CC) at day +100 was not different in HLA-identical compared to haploidentical HCT (32.5% and 38.2%, *P*=0.054). Post-HCT complications, including neutropenic fever (59.3% vs. 40%), acute graft-versus-host-disease (aGVHD) (46.9% vs. 25%), cytokine release syndrome (CRS) (18.5% vs. 2.5%), and hospitalization (71.6% vs 42.5%) were significantly more frequent in haploidentical HCT. Two-year overall survival (OS) was 60.6% vs. 46.9%, (*P*=0.464) for HLA-identical and haplo-HCT, respectively. There was no difference in the 2-year disease-free-survival (DFS) (33.3% vs. 35%, *P*=0.924) between transplant types. In multivariate analysis, positive measurable residual disease (MRD) at 30 days (HR 8.8, *P*=0.018) and 100 days (HR 28.5, *P*=0.022) was associated with lower OS, but not with non-relapse mortality (NRM) (*P*=0.252 and *P*=0.123, univariate). In univariate analysis, both 30-day and 100-day MRD were associated with lower DFS rates (*P*=0.026 and *P*=0.006), but only day 30 MRD was significant in multivariate analysis (*P*=0.050). In the case of relapse, only MRD at day 100 was associated with increased risk in the univariate and multivariate analyses (HR 4.48, *P*=0.003 and HR 4.67, *P*=0.008). Chronic graft-versus-host-disease (cGVHD) was protective for NRM (HR 0.38, *P*=0.015). There was no difference in cumulative incidence of relapse (CIR) between transplant types (*P*=0.126). Forty-four (36.4%) patients died, with no difference between HCT type (*P*=0.307). Septic shock was the most frequent cause of death with 17 cases, with no difference between transplant types

**Conclusions:**

Outpatient peripheral blood allogenic HCT after RIC is a valid and effective alternative for adult patients suffering acute myeloblastic or lymphoblastic leukemia in low-income populations.

## 1 Introduction

Allogeneic hematopoietic cell transplantation (allo-HCT) is a potentially curative option for patients with acute leukemia with previous failed treatments (1). Improved criteria for selection and better transplant strategies have balanced outcomes between HLA-identical and haploidentical allografting (2). With haploidentical transplantation, virtually all patients have a potential donor (3). Reduced-intensity conditioning (RIC) and improved GVHD prophylaxis are associated with fewer toxic-related complications, allowing for an outpatient setting (4). This scenario considerably reduces costs and is highly relevant for low-middle income countries (LMIC) lacking optimally funded health systems, a well-established donor network, and fully equipped and staffed transplant units (5).

An outpatient setting broadens HCT, providing access to many uninsured patients who would otherwise die from disease progression; its efficacy in a LMIC has been described (6). In this situation, acute myeloid leukemia (AML) and acute lymphoblastic leukemia (ALL) have been reported to be more prevalent in Latin American populations compared to developed countries (7, 8). Few studies report outcomes after an outpatient allo-HCT strategy employing a RIC scheme in LMIC as treatment for acute leukemia (6). This information is needed to allow physicians to identify the benefits, risks, and limitations of employing this transplant modality.

This study assessed the efficacy and safety of outpatient allo-HCT with a RIC regimen performed in a LMIC in patients with ALL or AML. We report patients’ clinical characteristics, most frequent post-HCT complications, clinical evolution, survival and relapse rates, and risk factors associated with mortality and relapse between donor types.

## 2 Materials and methods

### 2.1 Study design and patients

This single-center retrospective cohort study included patients ≥ 18 years diagnosed with AML or ALL who underwent an outpatient allo-HCT after RIC at the Hematology Department of the Dr. José E. González University Hospital of the School of Medicine of the Autonomous University of Nuevo León, in Monterrey, Mexico, from 2012 to 2022. Patients who had complete information on the clinical file and electronic database, and who received the conditioning regimen, central venous catheter insertion, and hematoprogenitors graft infusion in the ambulatory setting were included. Eligibility involved a Karnofsky score ≥70%, creatinine <2 mg/dL, ECOG ≤2, HCT-specific comorbidity index ≤2, and round-the-clock availability of a caregiver for the first month after HCT. The stem cell source was non-T-cell depleted peripheral blood; the selection of HLA-identical or haplo-HCT depended on the availability of the corresponding sibling donor. Our center cares for an open population, mainly low-income uninsured patients from the country’s northeast region. This study received approval by the institutional ethics and research committees (HE21-00033), and the principles of the Helsinki Declaration were observed. All patients provided written informed consent before transplantation.

### 2.2 Donors mobilization, collection, and stem cell source

Granulocyte colony-stimulating factor at 10 μg/kg/day was administered subcutaneously to donors for 4 days. Peripheral blood hematopoietic stem cells were collected through peripheral veins, or a central venous catheter placed in the outpatient clinic on the 5^th^ day. Single large volume leukapheresis processing was 3 to 5 times the estimated donor’s total blood volume to obtain at least 4x10^6^/kg viable CD34+ cells. The peripheral blood T-cell repleted graft was refrigerated and infused unmanipulated the same day of its collection.

### 2.3 Outpatient allo-HCT procedure and criteria for hospital admission

A detailed description of the outpatient HCT methods and transplant unit functioning has been previously described (9). All transplants were performed in an outpatient setting, including conditioning regimen administration, central catheter placement, stem cell infusion, and posttransplant cyclophosphamide (PTCY) administration. Patients who experienced regimen-related toxicity, grade III-IV hemorrhagic cystitis, severe mucositis, oral intolerance, diarrhea, severe neutropenic fever, hemodynamic instability or cardiovascular complications, severe cytokine release syndrome (CRS), or severe infection were hospitalized.

### 2.4 Outpatient supportive care

After graft infusion in the outpatient hematology clinic, patients went home under the supervision of their caregivers who had been instructed regarding specific needs; the nursing staff provided instructions on hygiene measures for the patient and caregiver. The treating physician explained the required medications, their presentation, dose, and schedule, and detection and course of action in case of untoward reactions, untoward effects, changes in psychological well-being, and signs of emergency events requiring immediate attention. Round-the-clock contact numbers were provided to seek urgent attention at any time and day. Staying at home following strict hygiene measures and diet indications was required during the first four post-transplant weeks. Patients had daily outpatient follow-up visits at the hematology day clinic from day +1 to +4, then every 48 hours until hematological recovery was observed. Subsequent visits were scheduled weekly until day +90, and then every month, or as frequently as required by the posttransplant clinical evolution. (10) Long-term follow-up for outstate recipients consisted of appointments at the hematology clinic every 2-3 months for two years, with monthly interim visits to the patient´s local physician.

### 2.5 Conditioning regimens and graft-versus-host disease prophylaxis

HLA-identical recipients received intravenous cyclophosphamide (CY) 350 mg/m^2^ and fludarabine (Flu) 25 mg/m^2^ from day -7 to -5, followed by oral busulphan (Bu) 4 mg/kg on days -4 to -2, or melphalan 140-200 mg/m^2^ i.v. on day -1. For graft-versus-host disease (GVHD) prophylaxis, oral cyclosporine (CsA) 5 mg/kg was employed on day -1, and intravenous methotrexate (5mg/m^2^) on days +1, +3, and +5. Oral CsA was continued through day +120-180 and then individualized, adjusting for a target level of 150-250 ng/mL and tapered over 30-60 days. If GvHD was suspected, oral CsA was tapered for a longer period.

Conditioning for haploidentical recipients included CY (350 mg/m^2^) and Flu (25 mg/m^2^), which were administered from days -4 to -2, and melphalan (MEL) (140-200 mg/m^2^) on day -1 with or without 2 Gy of total body irradiation. GvHD prophylaxis consisted of PT-CY (50 mg/kg/day) administered on days +3 and +4, mycophenolic acid (2 g/day) from day +5 to day +30, and oral CsA with the same dosing scheme described for the HLA-identical group. The stem cell source was unmanipulated peripheral blood and was infused on day 0.

Acute GVHD (aGVHD) was graded according to the Glucksberg criteria, and chronic (cGVHD) according to the National Institutes of Health criteria (11, 12).

### 2.6 Chimerism and measurable residual disease

Chimerism was determined by PCR analyses of short tandem repeats or variable number tandem repeats. In the case of sex mismatch between donor and recipient, chimerism was assessed by fluorescence *in situ* hybridization. The analysis was performed on days 30, 100, and 365 post-HCT. Complete chimerism required a ≥95% identical result between donor and patient in variable number tandem repeats, short tandem repeats, or fluorescence *in situ* hybridization analysis.

### 2.7 Antimicrobial prophylaxis

Anti-infectious prophylaxis initiated on day 0 with an oral quinolone (Levofloxacin 500 mg/day), oral itraconazole (100 mg/day PO BID), or voriconazole 200 mg twice a day, and daily low-dose oral acyclovir (800 mg/day) until engraftment. Subsequently, trimethoprim-sulfamethoxazole 160/800 mg/day 3 days per week and daily low-dose oral acyclovir (800 mg/day) were administered until immunosuppressive therapy was halted. Meropenem i.v., 1 gram every 8 hours, was administered empirically for neutropenic fever, unless resistant bacteria were documented; in that case targeted antibiotic combinations were employed. Cytomegalovirus (CMV) viral load was determined at days 14 and 30 post-transplant for all patients and further evaluated on a clinical basis according to their risk factors, such as GVHD development, corticosteroid use, prior reactivation, or when suspected by the treating physician. CMV infection treatment consisted of valganciclovir (450 mg BID) at a ≥500 IU/mL threshold. A different donor was chosen in the case of second transplants; the conditioning regimen was the same as in the first procedure.

### 2.8 Definitions

The transplant was considered ambulatory if the conditioning regimen and stem cell infusion were performed completely in the outpatient clinic, regardless of whether the patient required post-transplant hospitalization. Overall survival (OS) was defined as the time from the allo-HCT to death or last follow-up. Disease-free survival (DFS) was defined as the time from the allo-HCT to GF, disease progression, relapse, death, or last follow-up, whichever occurred first. Non-relapse mortality (NRM) was defined as death from any cause without relapse. Transplant-related mortality (TRM) was defined as death due to any transplant-related cause other than disease relapse or progression. Transplant-related hospitalization was any transplant-related event requiring inpatient care within day +1 to day +100 in the post-HSCT period. Neutrophil engraftment was defined as an absolute neutrophil count (ANC) of >0.5 x 10^9^/L for at least three consecutive days unsupported by granulocyte colony-stimulating factor. Platelet engraftment was when platelets reached >20 x 10^9^/L for at least 7 consecutive days without transfusion support. Primary graft failure (GF) was defined as neutrophil engraftment failure by day +28 and secondary GF was defined as a drop in ANC <0.5 x 10^9^/L after initial engraftment with loss of donor chimerism. Complete chimerism was assumed if ≥95% of hematopoietic cells were from donor origin; mixed chimerism was defined as 5 to 95% donor cells and the absence of chimerism as <5% donor cells (13). In the case of second transplants, only the last procedure was included, and it was assessed the same way as the first transplants.

### 2.9 Statistical analysis

SPSS version 25 (IBM Corp., Armonk, NY) was used for all data analysis, except cumulative incidence analyses that were estimated using R (Murray Hill, NJ, USA). A descriptive analysis used medians (range) for continuous variables and proportions for categorical variables. Univariate comparison of basal characteristics and HCT complications was conducted between donor types using the chi-square test for categorical variables or the Mann-Whitney test for continuous variables. Overall survival and DFS were estimated with Kaplan-Meier curves and compared between groups with the log-rank test. Cox proportional hazard regression analysis was performed to identify OS, DFS, NRM and relapse risk factors in univariate and multivariate analysis. For the multivariate analysis, stepwise analysis was used with a variable entry criterion of p <.1. Cox analyses were reported as the hazard ratio (HR), 95% confidence interval (CI) for the HR, and the corresponding p-value. The cumulative incidence of relapse was estimated from the time of allo-HCT to relapse/progression. The cumulative incidence of NRM was estimated from the time of allo-HCT to death or last follow-up without relapse/disease progression. As a secondary analysis, patients were analyzed stratified according to type of acute leukemia, lymphoblastic vs. myeloblastic. A p-value ≤.05 was considered statistically significant.

## 3 Results

### 3.1 Patient, donor, and allo-HCT characteristics

One hundred twenty-one outpatient allo-HCTs were performed during our study period; 81 (66.9%) were haploidentical, and 40 (33.1%) were HLA-identical. Diagnoses were ALL in 64 (52.9%) and AML in 57 (47.1%) patients. Nine (7.4%) patients underwent their second allo-HCT. Median CD34+ infused cells were 10x10^6^/kg for haploidentical and 8x10^6^/kg for HLA-identical recipients (*P*=0.006). Myeloid engraftment was achieved in a median of 14 days for both groups; platelet engraftment median time was 16 days for haploidentical and 14 days for HLA-identical recipients (*P*=0.013). 69.1% vs. 72.5% patients had reactive IgG anti-CMV in the haploidentical and HLA-identical HCT group, respectively (*P*=0.047). The median follow-up for the cohort was 31 months (10–98). Other demographic and clinical characteristics, including CMV status in recipient/donor pairs, are summarized in **Table 1**.

**Table 1:** Baseline characteristics of 121 adults with acute leukemia who underwent an outpatient allogeneic hematopoietic cell transplantation with a reduced intensity conditioning.

Variable	Total (n=121)	HLA-Identical (n=40)	Haploidentical (n=81)	p-value
Recipient age, median (range)	28 (18–68)	35 (18–64)	26 (18–68)	.122
Donor age, median (range)	35 (18-66)	37 (18-66)	32 (18-61)	.107
Gender, n (%)				.273
Male	69 (57)	20 (50)	49 (60.5)	
Female	52 (43)	20 (50)	32 (39.5)	
Diagnosis				.138
ALL	64 (52.9)	17 (42.5)	47 (58)	
AML	57 (47.1)	23 (57.5)	34 (42)	
Second HCT	9 (7.4)	5 (12.5)	4 (4.9)	.136
Infused CD34+ cells x 10^6^/kg, median (range)	9.5 (3-15)	8 (3-13)	10 (4-15)	.006
Time from diagnosis to HCT in months, median (range)	13.5 (1-132)	20 (1-132)	11 (1-96)	.098
Engraftment in days, median (range)				
Myeloid	14 (10-25)	14 (10-20)	14 (11-25)	.464
Platelets	15 (11-30)	14 (11-21)	16 (11-30)	.013
Donor/receptor sex match				
Male/Male	41 (33.9)	12 (30.8)	29 (35.4)	.691
Male/Female	22 (18.2)	8 (20.5)	14 (17.1)	.591
Female/Male	23 (19)	6 (15.4)	17 (20.7)	.526
Female/Female	29 (24)	11 (28)	18 (22)	.588
ABO incompatibility ^a^	38 (30.6)	14 (34.2)	24 (29.7)	.489
Major ABO incompatibility	19 (15.7)	6 (15.4)	13 (15.9)	.926
Minor ABO incompatibility	20 (15.7)	8 (17.9)	12 (14.6)	.433
Recipient/donor CMV serostatus				
Positive/positive	68 (56.2)	22 (55)	46 (56.7)	.264
Positive/negative	17 (14)	7 (17.5)	10 (12.3)	.262
Negative/positive	11 (9.1)	0	11 (13.5)	.032
Negative/negative	10 (8.3)	1 (2.5)	9 (11.1)	.277
Follow-up time in months, median (range)	31 (10-98)	61 (2-98)	22 (1-69)	.254

a One patient had bidirectional ABO incompatibility.

ALL, acute lymphoblastic leukemia; AML, acute myeloblastic leukemia; HCT, hematopoietic cell transplantation; CMV, cytomegalovirus.

The salient characteristics of patients in the cohort according to type of acute leukemia are shown in **Table 2**. Patients were younger in the ALL group, median age of 19 vs. 38 years in the AML group (*P*=0.001), median time from diagnosis to transplant was considerably longer in ALL patients, 15 vs. 8 months in AML patients (*P*=0.001); also, median CD34+ cell dose infused was higher for ALL recipients, 10x10^6^/kg of body weight vs. 8.8x10^6^/kg in AML cases (*P*=0.024). Regarding clinical features, only cytokine release syndrome (CRS) incidence was higher in ALL patients, 20.3% vs. 5.3% (*P=*0.015) in AML patients. Mortality rate according to leukemia type was, for the ALL group, 34.4% vs. 38.6% for AML patients, (*P*=0.630), while 50% vs. 57.9% (*P*=0.385), suffered relapse, progression, or graft failure, respectively.

**Table 2:** Comparison of salient characteristics and main outcomes between 64 patients with ALL and 57 with AML who underwent an outpatient hematopoietic cell transplantation with a reduced intensity conditioning.

Variable	Total (n=121)	ALL (n=64)	AML (n= 57)	p-value
Recipient age, median (range)	28 (18-68)	19 (18-64)	38 (18-68)	0.001
Type of transplant, n (%)				0.108
Haploidentical	81 (66.9)	47 (73.4)	34 (59.6)	
HLA-identical	40 (33.1)	17 (26.6)	23 (40.4)	
Time from diagnosis to HCT in months, median (range)	13.5 (1-132)	15 (3-132)	8 (1-105)	0.001
Infused CD34+ cells x 10^6^/kg, median (range)	9.5 (2.9-15)	10 (3.9-15)	8.8 (2.9-15)	0.024
Engraftment in days, median (range)				
Myeloid	14 (10-25)	15 (10-25)	14 (11-21)	0.207
Platelets	15 (11-30)	15 (11-30)	15 (11-26)	0.057
aGVHD, n (%)	48 (39.7)	27 (42.2)	21 (39.8)	0.549
cGVHD, n (%)	28 (23.1)	14 (21.9)	14 (24.6)	0.727
CRS, n (%)	16 (13.2)	13 (20.3)	3 (5.3)	0.015
Hospital admission, n (%)	75 (62)	40 (62.5)	35 (61.4)	0.901
Days of hospitalization, median (range)	11(1-54)	10.5 (1-54)	12 (1-48)	0.445
# of blood products, median (range)	2 (1-13)	1 (1-12)	3 (1-13)	0.130
# of platelet concentrates, median (range)	11 (1-63)	10 (3-61)	12 (1-63)	0.483
Relapse, n (%)	41 (33.9)	18 (28.1)	23 (40.4)	0.128
Mortality, n (%)	44 (36.4)	22 (34.4)	22 (38.6)	0.630
Chimerism day 30 ^a^				
Complete chimerism	67 (55.3)	35 (54.7)	32 (56.1)	0.479
Mixed chimerism	21 (17.4)	12 (18.8)	9 (15.8)	0.362
Absent chimerism	2 (1.7)	2 (3.1)	–	0.109
Chimerism day 100 ^b^				
Complete chimerism	44 (36.4)	22 (34.4)	22 (38.6)	0.426
Mixed chimerism	17 (14)	11 (17.2)	6 (10.5)	0.274
MRD day 30 a, n (%)				0.072
Positive	6 (4.9)	5 (7.8)	1 (1.8)	
Negative	73 (60.3)	33 (51.6)	40 (70.2)	
MRD day 100^b^, n (%)				0.803
Positive	13 (10.7)	7 (10.9)	6 (10.5)	
Negative	56 (46.2)	28 (43.8)	28 (49.1)	

a Chimerism was unknown for 7 (17.5%) HLA-identical and 23 (28.5%) haploidentical recipients. MRD was unknown for 19 (47.5%) HLA-identical and 23 (28.5%) haploidentical recipients.

b Chimerism was unknown for 17 (42.5%) HLA-identical and 43 (53%) haploidentical recipients. MRD was unknown for 17 (42.5%) HLA-identical and 35 (43.2%) haploidentical recipients.

ALL, acute lymphoblastic leukemia; AML, acute myeloblastic leukemia; HCT, hematopoietic cell transplantation; aGVHD, acute graft-versus-host disease; cGVHD, chronic graft-versus-host disease; CRS, cytokine release syndrome; MRD, measurable residual disease.

### 3.2 Allo-HCT outcomes and complications

Forty-eight (59.3%) and 16 (40%) patients had neutropenic fever in haploidentical and HLA-identical HCT, respectively (*P*=0.027). Infections occurred in 53 (43.8%) patients, bacteria being the most frequent cause in 30 (56.6%), *E. coli* being the most frequent agent in 27% of the cases. aGVHD incidence was lower in HLA-identical (25%) than haplo-HCT (46.9%), *P*=0.016. On the other hand, cGVHD was not different between haploidentical and HLA-identical HCT, 25.9% and 17.5%, respectively (*P*=0.251). Cytokine release syndrome was present in 18.5% of haploidentical and 2.5% of HLA-identical recipients (*P*=0.020). Cytokine release syndrome grade was similar between groups (*P*=0.108).

All transplants were outpatient, yet 58 (71.6%) haploidentical recipients were hospitalized compared to 17 (42.5%) HLA-identical recipients (*P*=0.002). The most common cause of hospitalization was neutropenic fever (58.6%), with no difference observed between groups. Seventeen (14%) patients required more than 1 hospitalization, with no difference between HCT type. There was a difference in days of hospital stay between HLA-identical and haploidentical recipients, 8 (1–23) vs. 12 (1–54) days respectively (*P*=0.024). No difference was observed at 30 and 100-day chimerism between HCT types. MRD screening at 30 and 100 days was similar between groups. A detailed description of the allo-HCT outcomes and complications is shown in **Table 3**.

**Table 3:** Outcomes after outpatient allogeneic hematopoietic cell transplant (HCT) in 121 adults with acute leukemia.

Variable, n (%)	Total, n=121	HLA-Identical, n=40	Haploidentical, n=81	p-value
Neutropenic fever	64 (52.9)	16 (40)	48 (59.3)	.027
Infections (any type)	53 (43.8)	14 (35)	39 (48.1)	.170
Infections				.477
Bacterial	30 (56.6)	7 (53.8)	23 (60.5)	
Fungal	11 (20.8)	3 (23.1)	8 (21.1)	
Viral	10 (18.9)	3 (23.1)	7 (18.4)	
COVID-19 ^a^	5 (4.1)	1 (2.5)	4 (4.9)	.526
CMV reactivation	36 (29.8)	9 (22.5)	27 (33.3)	.208
aGVHD	48 (39.7)	10 (25)	38 (46.9)	.016
cGVHD	28 (23.1)	7 (17.5)	21 (25.9)	.251
CRS	16 (13.2)	1 (2.5)	15 (18.5)	.020
CRS grade				.108
Grade 1	12 (75)	1 (100)	11 (73.3)	
Grade 2	3 (18.8)	0 (0)	3 (20)	
Grade 3	1 (6.2)	0 (0)	1 (6.6)	
Mucositis	25 (20.7)	8 (20)	17 (21)	.873
Mucositis grade				.959
Grade 1	13 (52)	4 (50)	9 (53)	
Grade 2	6 (24)	2 (25)	4 (23.5)	
Grade 3	5 (20)	2 (25)	3 (17.6)	
Grade 4	1 (4)	0 (0)	1 (5.9)	
Hospital admission	75 (62)	17 (42.5)	58 (71.6)	.002
Causes of hospitalization, n=75				.196
CRS	2 (2.7)	0 (0)	2 (2.7)	
Infection	6 (8.0)	3 (4)	3 (4)	
Neutropenic fever	44 (58.6)	10 (13.3)	34 (44.7)	
Septic shock	6 (8.0)	1 (1.3)	5 (6.7)	
Other	17 (22.7)	5 (6.7)	12 (16)	
Days of hospitalization, median (range)	11 (1-54)	8 (1-23)	12 (1-54)	.024
>1 hospital admission	17 (14)	4 (18.2)	13 (16)	.206
# of blood products, median (range)	2 (1-13)	1.5 (1-13)	2 (1-12)	.999
# of platelet concentrates, median (range)	11 (1-63)	10 (5-36)	12 (3-63)	.322
Graft failure	10 (8.2)	3 (7.5)	7 (8.6)	.847
Relapse	41 (33.9)	16 (40)	25 (30.9)	.447
Disease progression	5 (4.1)	2 (5)	3 (3.7)	.793
Mortality	44 (36.4)	12 (30)	32 (39.5)	.307
Main causes of mortality, n=44				.423
Septic shock	17 (38.6)	3 (6.8)	14 (31.8)	
Respiratory insufficiency/infection	6 (13.6)	3 (6.8)	3 (6.8)	
Relapse/Progression	13 (29.5)	4 (9.1)	9 (20.5)	
Cardiogenic shock	2 (4.5)	1 (2.3)	1 (2.3)	
Chimerism day 30 ^b^				
Complete chimerism	67 (55.4)	19 (47.5)	48 (59.2)	.473
Mixed chimerism	21 (17.3)	13 (32.5)	8 (9.9)	.253
Absent chimerism	3 (2.4)	1 (2.5)	2 (2.4)	.955
Chimerism day 100 ^c^				
Complete chimerism	44 (36.4)	13 (32.5)	31 (38.2)	.054
Mixed chimerism	17 (14)	10 (25)	7 (8.6)	.059
MRD day 30 ^b^				.567
Positive	6 (4.9)	1 (2.5)	5 (6.1)	
Negative	73 (60.3)	20 (50)	53 (65.4)	
MRD day 100 ^c^				.276
Positive	13 (10.7)	6 (15)	7 (8.6)	
Negative	56 (46.2)	17 (42.5)	39 (48.1)	

a Considering HCTs performed since 2020 (n=51): HLA-identical (n=11) and haploidentical (n=40).

b Chimerism was unknown for 7 (17.5%) HLA-identical and 23 (28.5%) haploidentical recipients. MRD was unknown for 19 (47.5%) HLA-identical and 23 (28.5%) haploidentical recipients.

c Chimerism was unknown for 17 (42.5%) HLA-identical and 43 (53%) haploidentical recipients. MRD was unknown for 17 (42.5%) HLA-identical and 35 (43.2%) haploidentical recipients.

CMV, cytomegalovirus; aGVHD, acute graft-versus-host disease; cGVHD, chronic graft-versus-host disease; CRS, cytokine release syndrome; MRD, measurable residual disease.

### 3.3 Survival outcomes

**Figure 1:**
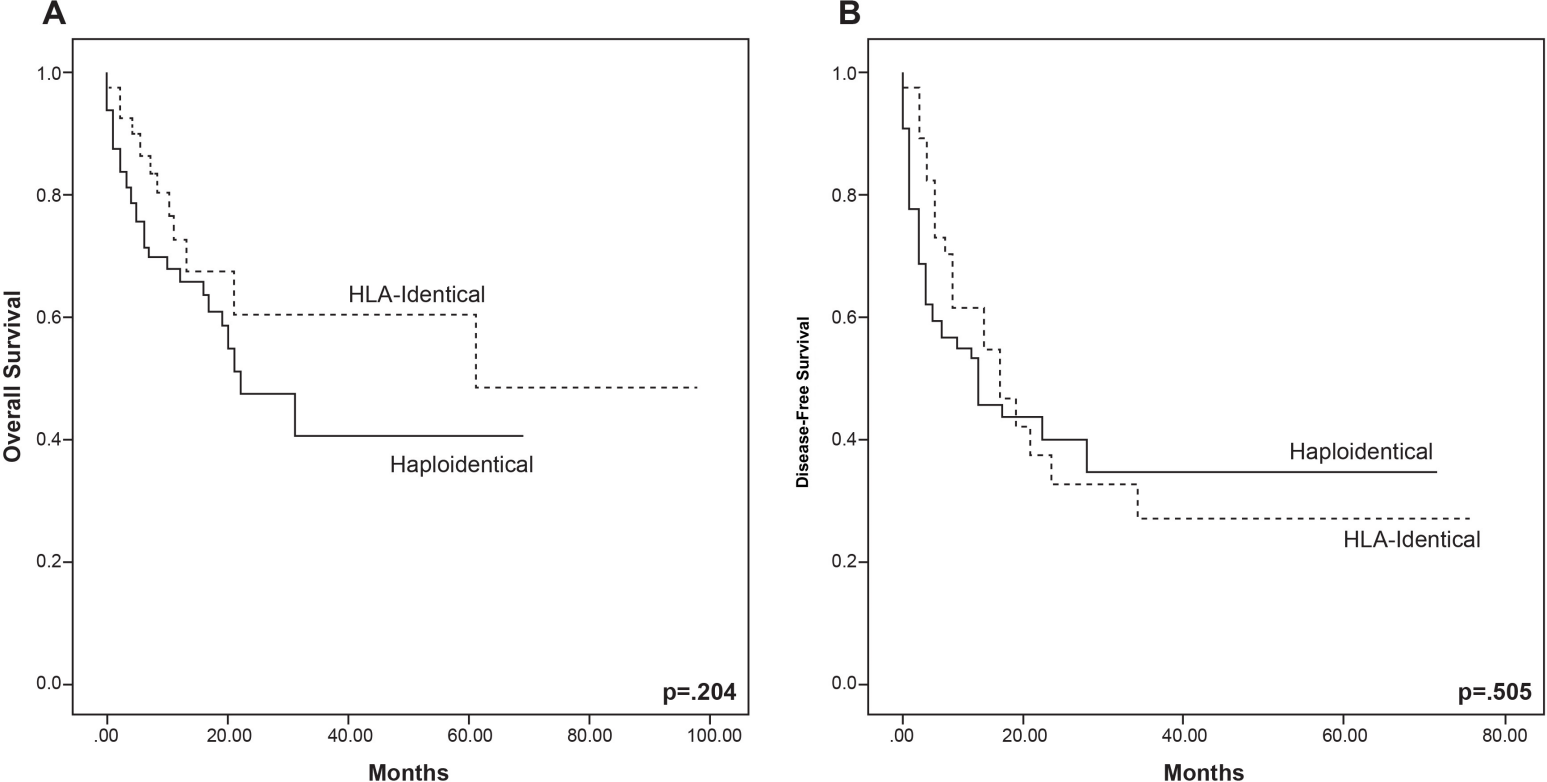
Overall survival **(A)** and disease-free survival **(B)** by allogeneic hematopoietic cell transplant types of 121 patients with acute leukemia in a completely outpatient setting and with reduced intensity conditioning. P-values represent overall comparisons without reference to specific time points.

**Figure 2:**
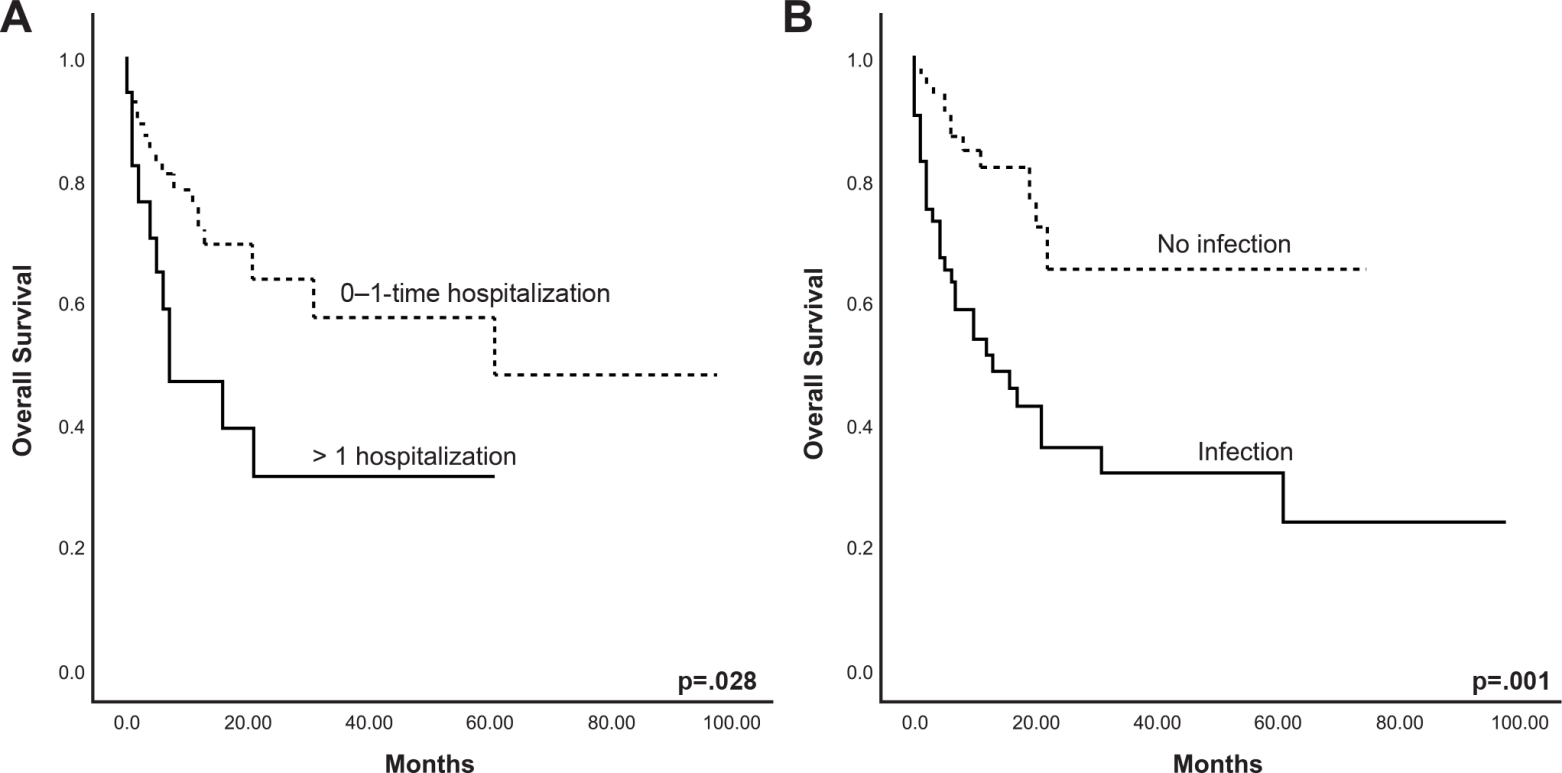
Overall survival of 121 patients with acute leukemia considering those who presented post-hematopoietic cell transplant complications: **(A)** >1 hospitalization, **(B)** infection. P-values represent overall comparisons without reference to specific time points.

At 31 months 50% of the cohort was alive, median OS for HLA-identical recipients was 61 (2–98) months, while for haplo-HCT it was 22 (1–69) months (*P*=0.283). The 2-year OS was 60.6% and 46.9% for HLA-identical and haploidentical HCT, respectively (*P*=0.464), OS for the whole cohort is shown in **Figure 1A**. Forty-four (36.4%) patients died in a median time of 6 (1–61) months, with no difference between HCT type (P=0.307). The main causes of death were septic shock (n=17, 38.6%), relapse or progression (n=13, 29.5%), and respiratory insufficiency or infection (n=6, 13.6%). Five-year OS for the patients diagnosed with ALL was 49.4%, while in the AML group it was 46.2% (*P*=0.508). Univariate and multivariate Cox regression analyses for OS were estimated (**Table 4**). In univariate and multivariate analysis, positive MRD at 30 days (HR 4.18, *P*=0.011 and HR 8.8, *P*=0.018) and 100 days (HR 4.72, *P*=0.003 and HR 28.5, *P*=0.022) was associated with lower OS, while in the univariate analysis, patients with infections of any type had an increased risk for an event in the OS (HR 3.28, *P*=0.001); bacterial infections were of most risk (HR 2.41, *P*=0.005). These findings are illustrated in **Figure 2**. Median OS for patients with 30-day positive MRD was 12 months, versus 61 months for those negative (*P*=0.005).

**Table 4:** Univariate and multivariate analyses of risk factors for Overall Survival and Disease-Free Survival in acute leukemia after an outpatient allogeneic hematopoietic cell transplant with a reduced intensity regimen.

Variable	OS				DFS			
	Univariate		Multivariate		Univariate		Multivariate	
	HR (95% CI)	p	HR (95% CI)	p	HR (95% CI)	p	HR (95% CI)	p
Age	1.01 (0.98-1.03)	.137			1.01 (0.99-1.02)	.099		
Gender (male)	1.34 (0.72-2.48)	.350			1.24 (0.75-2.05)	.383		
HCT type (Haploidentical)	1.50 (0.78-2.98)	.212			1.18 (0.70-1.98)	.519		
Infused CD34+ cells x 10^6^/kg	0.98 (0.88-1.09)	.752			1.05 (0.92-1.09)	.900		
Neutropenic fever	1.77 (0.95-3.29)	.067			2.01 (1.20-3.37)	.008	1.29 (0.18-8.95)	.794
Infections (any type)	3.28 (1.71-6.29)	.001	0.12 (.03-5.48)	.124	2.21 (1.35-3.63)	.002	1.31 (0.16-10.42)	.793
Bacterial infection	2.41 (1.31-4.43)	.005	1.81 (.01-284.19)	.818	1.81 (1.08-3.01)	.025	3.14 (0.32-30.49)	.323
aGVHD	0.70 (0.37-1.30)	.264			0.54 (0.32-0.92)	.023	0.43 (0.08-2.23)	.318
cGVHD	0.48 (0.21-1.06)	.071			0.42 (0.21-0.83)	.013	1.98 (1.17-9.92)	.477
CRS	0.68 (0.24-1.98)	.466			0.90 (0.40-1.99)	.799		
Hospital admission	1.63 (0.80-3.33	.177			1.49 (0.85-2.61)	.162		
>1 hospital admission	2.27 (1.06-4.85)	.035	5.80 (0.46-73.15)	.174	2.39 (1.24-4.59)	.009	9.55 (1.54- 59.05)	.015
RBC transfusion	2.45 (1.09-5.51)	.029	11.98 (0.74-194.15)	.08	2.02 (1.08-3.77)	.027	6.12 (2.85- 31.8)	.247
Platelet transfusion	0.60 (0.20-1.76)	.356			0.95 (0.33-2.69)	.929		
Recipient-positive IgG anti-CMV	1.54 (0.64-3.96)	.329			2.75 (1.18-6.42)	.019	(10.28 3.83-23.42)	.098
CC at 30 days	0.95 (0.31-2.93)	.936			0.78 (0.26-2.29)	.654		
CC at 100 days	0.72 (0.23-2.30)	.589			0.71 (0.29-2.19)	.562		
MRD at 30 days	4.18 (1.28-12.58)	.011	8.80 (1.44- 53.73)	.018	2.95 (1.13-7.65)	.026	22.56 (2.53- 200)	.050
MRD at 100 days	4.72 (1.83-12.16)	.003	28.54 (1.61-50.55	.022	5.27 (1.60-17.13)	.006	12.9 (0.92-18.13)	.054

HCT, hematopoietic cell transplantation; aGVHD, acute graft-versus-host disease; cGVHD, chronic graft-versus-host disease; CRS, cytokine release syndrome; CMV, cytomegalovirus; CC, complete chimerism; MRD, measurable residual disease.

Although hospital admissions *per se* had no risk for mortality, patients with more than 1 hospitalization were at greater risk (HR 2.27, *P*=0.035). Patients who received red blood cell transfusions (HR 2.45, *P*=0.029) had an increased mortality risk in univariate analysis. Platelet transfusions did not increase this risk, **Table 4**.

### 3.4 Disease-free survival

**Figure 3:**
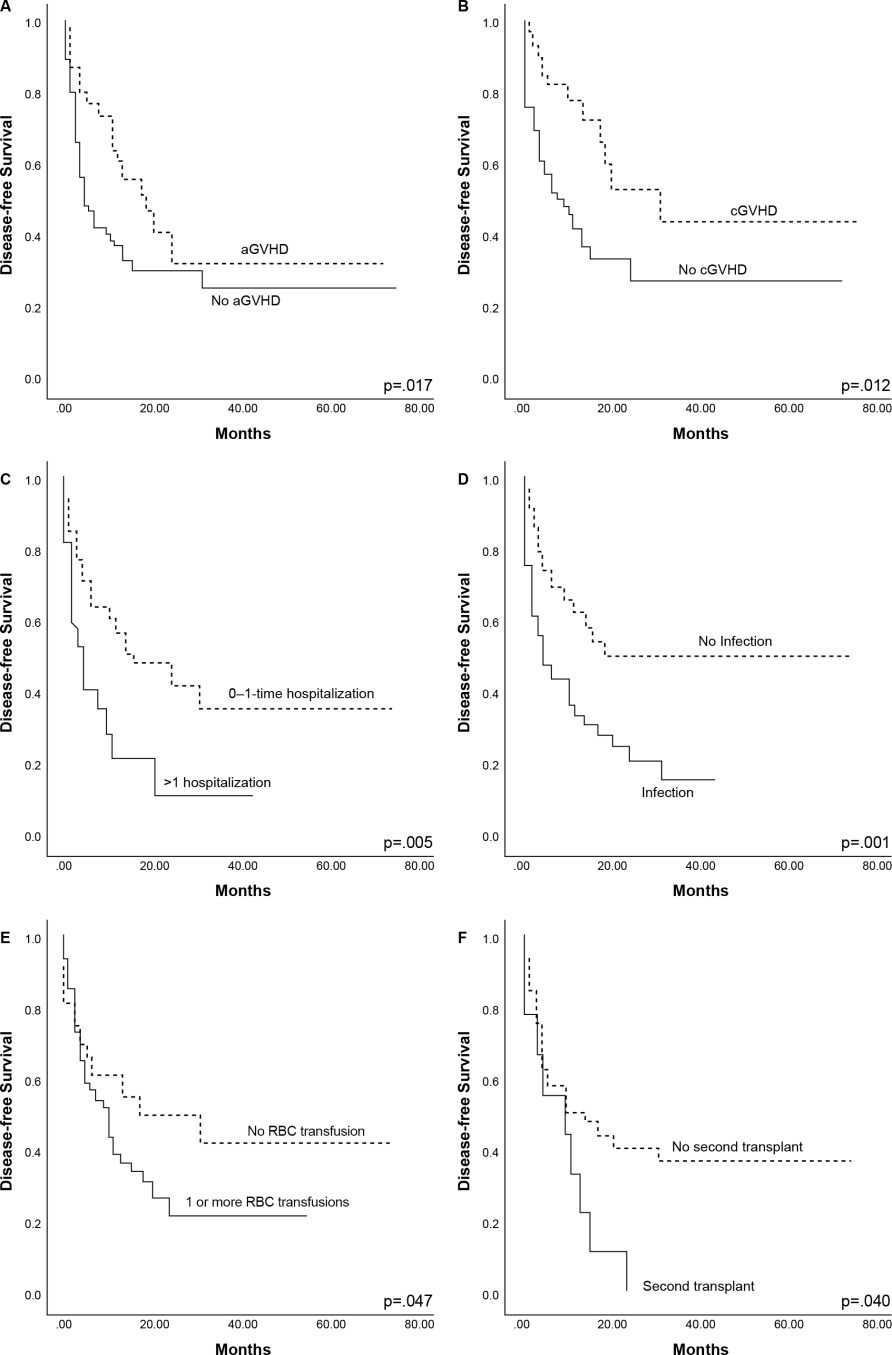
Complications affecting disease-free survival of 121 patients with acute leukemia: **(A)** acute graft-versus-host-disease (aGVHD), **(B)** chronic graft-versus-host-disease (cGVHD), **(C)** >1 hospitalization, **(D)** infection, **(E)** transfusion, **(F)** second transplant. P-values represent overall comparisons without reference to specific time points.

The median DFS for the total cohort was 6 (1–75) months, 9 (1–75) months for HLA-identical, and 5 (1–69) for haplo-HCT (*P*=0.067). The two-year DFS was 33.3% and 35% for HLA-identical and haplo-HCT, respectively (*P*=0.924), **Figure 1B**. Forty-one (33.9%) patients relapsed, 5 (4.1%) had disease progression, and 10 (8.2%) had GF after allo-HCT, with no difference between HCT types for any of these events (**Table 3**). Cox regression analyses for DFS are shown in **Table 4**. In the univariate analysis, post-HCT complications, such as neutropenic fever (HR 2.01, *P*=0.008), infections of any type (HR 2.21, *P*=0.002), bacterial infections (HR 1.81, *P*=0.025), more than 1 hospitalization (HR 2.39, *P*=0.009), and RBC transfusions (HR 2.02, *P*=0.027), were all associated with increased risk of lower DFS. Both aGVHD (HR 0.540, *P*=0.023) and cGVHD (HR 0.42, *P*=0.013) conferred a lower risk for an event in DFS in the univariate analysis, this was confirmed in Kaplan-Meier analysis, with a higher DFS in the presence of aGVHD (*P*=0.017) and cGVHD (*P*=0.012), **Figure 3A, B**. Recipient IgG reactive to CMV was a risk factor for lower DFS (HR 2.75, *P*=0.019). In univariate analysis, positive MRD at 30 (HR 2.95, *P*=0.026) and 100 days (HR 5.27, *P*=0.006) was associated with lower DFS. In the multivariate analysis, having >1 hospital admission (HR 9.55, P=0.015) and positive MRD on day 30 (HR 22.56, P=0.50) were associated with an increased risk for lower DFS. In the Kaplan-Meier analyses, >1 hospitalization, infection, red blood cell transfusion, and receiving a second transplant were all statistically significant for lower DFS, shown in **Figures 3C–F**. In multivariate analysis, only patients who had >1 hospital admission had lower DFS (HR 9.55, *P*=0.015).

### 3.5 Relapse and non-relapse mortality

**Figure 4:**
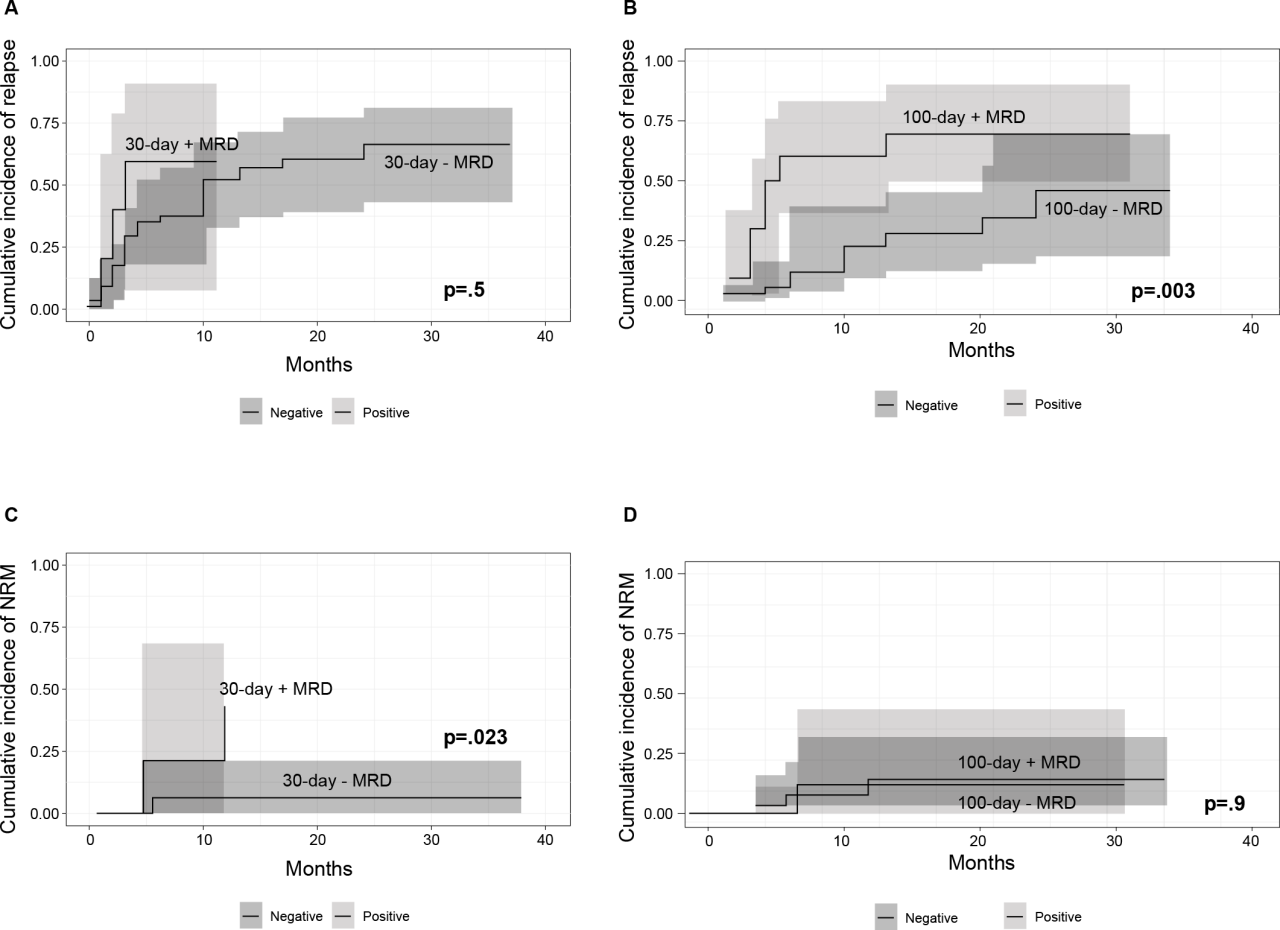
**(A)** Cumulative incidence of relapse (CIR) in patients with 30-day positive and negative minimal-residual disease (MRD) **(B)** CIR in patients with 100-day positive and negative MRD **(C)** CI of non-relapse mortality (NRM) in patients with 30-day positive and negative MRD **(D)** CI of NRM in patients with 100-day positive and negative MRD. P values refer to 6-month cumulative incidence of relapse and non-relapse mortality.

The cumulative incidence of relapse (CIR) for the whole cohort was 44% at 2 years (95% CI 32-55%). No significant difference was observed between transplant types (*P*=0.800). The cumulative incidence of NRM was 20% at 2 years (95% CI 13-28%) for the entire cohort, with no statistical difference between transplant types (*P*=0.500). Patients with positive 30-day MRD showed no difference in the 6-month CIR compared to those with negative 30-day MRD, 60% versus 38% (*P*=0.500), **Figure 4A**; yet a significant difference was observed in the 6-month CIR between positive and negative 100-day MRD, 60% versus 12% (*P*=0.003), **Figure 4B**. A significant difference was observed in the cumulative incidence of 6-month NRM between positive and negative MRD patients at day 30, 20% versus 6.3% (*P*=0.023), **Figure 4C**; this finding was not significant between the 100-day MRD groups (*P*=0.900), **Figure 4D**.

In the univariate analysis, an increase in age (HR 1.03, *P*=0.009) and patients with major ABO incompatibility (HR 2, *P*=0.040) had an increased risk for NRM. CGVHD was associated with decreased NRM in the univariate (HR 0.37, *P*=0.009) and multivariate analysis (HR 0.38, *P*=0.015). Positive MRD at 100 days increased relapse risk (HR 4.48, *P*=0.003) in the univariate and multivariate analyses (HR 4.67, *P*=0.008), as shown in **Table 5**.

**Table 5:** Univariate and multivariate analyses of risk factors for non-relapse mortality (NRM) and relapse in acute leukemia after an outpatient allogeneic hematopoietic cell transplant with a reduced intensity regimen.

Variable	NRM				Relapse			
	Univariate		Multivariate		Univariate		Multivariate	
	HR (95% CI)	p	HR (95% CI)	p	HR (95% CI)	p	HR (95% CI)	p
Age	1.03 (1.00-1.06)	.009	1.01 (0.99-1.03)	.064	0.99 (0.97-1.01)	.746		
Gender (male)	0.83 (0.34-2.04)	.692			1.06 (0.57-1.99)	.835		
HCT type (Haploidentical)	0.58 (0.22-1.50)	.264			0.99 (0.52-1.86)	.983		
Infused CD34+ cells x 10^6^/kg	0.90 (0.77-1.05)	.203			1.03 (0.93-1.14)	.482		
Major (ABO) incompatibility	2 (1.03-3.89)	.040	1.71 (0.66-4.40)	.263	1.89 (0.90-3.99)	.092		
Neutropenic fever	1.43 (0.83-2.44)	.188			1.68 (0.89-3.16)	.106		
Infections (any type)	0.87 (0.52-1.48)	.628			1.71 (0.92-3.17)	.084		
Bacterial infection	1.55 (0.89-2.67)	.115			0.84 (0.39-1.83)	.671		
RBC transfusion	0.88 (0.46-1.68)	.707			1.65 (0.79-3.42)	.180		
Platelet transfusion	1.01 (0.34-2.93)	.984			0.78 (0.23-2.63)	.693		
aGVHD	0.62 (0.36-1.08)	.096			0.62 (0.32-1.18)	.150		
cGVHD	0.37 (0.18-0.78)	.009	0.38 (0.19-0.83)	.015	0.71 (0.33-1.52)	.383		
CRS	1.92 (0.79-4.64)	.144			0.71 (0.25-2.02)	.527		
Hospital admission	1.15 (0.65-2.05)	.618			1.25 (0.63-2.48)	.513		
>1 hospital admission	1.46 (0.72-2.99)	.290			1.49 (0.61-3.60)	.373		
Recipient-positive IgG anti-CMV	1.36 (0.57-3.10)	.480			1.72 (0.67-4.46)	.258		
CC at 30 days	1.45 (0.46-4.56)	.525			0.84 (0.28-2.47)	.752		
CC at 100 days	.77 (0.23-2.53)	.668			0.69 (0.22-2.13)	.525		
MRD at 30 days	2.18 (0.57-8.27)	.252			3.03 (0.82-11.22)	.096		
MRD at 100 days	2.43 (0.78-7.51)	.123			4.48 (1.65-12.16)	.003	4.67 (1.77-12.33)	.008

HCT, hematopoietic cell transplantation; RBC, red blood cell; aGVHD, acute graft-versus-host disease; cGVHD, chronic graft-versus-host disease; CRS, cytokine release syndrome; CMV, cytomegalovirus; CC, complete chimerism; MRD, measurable residual disease.

## 4 Discussion

The introduction of RIC regimens and alternative stem cell sources has made HCT more available to treat acute leukemia when considered appropriate, such as in high-risk disease or after relapse. Two major advances in hematopoietic cell transplantation derived from Luznik and cols.’ pioneering work, namely, the use of high-dose post-transplant cyclophosphamide for GVHD prophylaxis and HLA-haploidentical allografting (14–16), benefited our cohort.

The two main causes of death in acute leukemia after an HCT are transplant-related complications or disease relapse. We found that our outpatient peripheral blood allogeneic HCT program with a RIC regimen for AML achieved a 2-year OS of 46.2%, DFS of 28.8%, and NRM of 27.5%, in comparison to 58.5%, 53.2%, and 33% at 3 years in AML patients, respectively, in one report (17); for our allografted adults with ALL the corresponding 2-year rates were OS 55.5%, DFS 39.6%, and NRM 17.8%, while in a comparable group, 3-year rates for OS were 66%, for DFS 72.4%, and for NRM 20.3% (18). In a study including 331 AML patients allografted using RIC, 2-year OS was 66%, and NRM 22.5% (19). Thus, our findings are important due to the limited data on post-HCT clinical outcomes for acute leukemia reported by transplant centers in low-middle income countries (LMIC). Several factors could help explain the lower DFS observed in our cohort in comparison to other reports, including a positive MRD at the time of transplant, a second transplant, the use of RIC in AML, the presence of undetected Ph+ cases in ALL patients, a low incidence of GVHD, and the administration of sequential conditioning regimens in chemo-resistant leukemias employing a toxic scheme. Since only RIC was used, HCT performed in our center was conducted in an outpatient setting, making of it a cost-effective method, with an estimated cost of $ 11,053 ± 2,817 USD for ALL, and 10,251 ± 1,538 USD for AML, as previously reported (5); these costs are considerably lower than those documented for RIC HSCT in a study carried out at two centers in the USA, ranging from 96,000 (74–152,000) to 129,000 (84–171,000) USD; no differences in costs were found between centers (20); lower mean costs are reported from other countries, including Spain, 76.112 USD (21). Importantly, the previous comparisons are made with studies using outpatient (USA) and home care (Spain) models, not hospitalization.

Additionally, most uninsured patients belonging to the open population cannot afford an inpatient HCT. Most of the HCTs performed in this study were haploidentical, partly favored by our country’s lack of a well-established donor network, a limitation shared with many other LMICs. As observed, most haploidentical HCTs received a higher CD34+ cell infusion and had slower platelet engraftment, in line with other reports comparing HLA-identical recipients (22, 23). A higher infusion dose of CD34+ cells is associated with earlier engraftment and increased survival in patients with acute leukemia (24, 25), although in our study no association between the infused dose and survival was observed. In this respect, it is worth mentioning that the establishment of a CD34+ cell dose threshold for improving HCT outcomes is controversial. Since the infusion dose can be controlled before employing the HCT, this association should be clarified in future studies.

Haploidentical HCT has been associated with poor immune recovery and infections that lead to higher mortality and relapse rates (26–28). In addition, in previous reports comparing outcomes for an HLA-identical sibling vs. HLA-haploidentical HCT, the haploidentical group had significantly higher NRM, lower progression-free and overall survival, and higher risk of viral and fungal infections, as well as other complications (29, 30).

In the present study, haploidentical HCTs had more neutropenic fever cases than HLA-identical transplants, yet it did not increase the mortality risk and was not associated either in the univariate or multivariate analyses with OS. Additionally, it is known that a serious and frequent complication of infection is neutropenic fever; infection has been shown to increase the risk for decreased OS (28). Our results show that the incidence of infections in HLA-identical and haploidentical HCT was similar, 35% and 48%, *P*=0.170. These findings show that the infection incidence is lower than that reported with myeloablative inpatient allogeneic HCT (31, 32). HCT with RIC produces less myelosuppression and mucosal toxicity, which are implicated in a higher infection risk due to microbiota imbalance (31). In our study, patients with infections had a significantly lower OS rate. Yet, no difference in OS was observed between HCT types, confirming that haplo-HCT represents a safe transplant alternative to HLA-identical allografting. Additionally, a RIC regimen allows transplantation to be conducted in an outpatient setting, which exposes the patient to fewer nosocomial infections and, thus, reduces the risk for increased morbidity.

Close to 45% of the patients in our series had a documented infection and almost 20% of the total suffered infection related mortality (IRM); in addition, in univariate analysis, OS and DFS were significantly lower in patients who suffered infection compared to those who did not. Thus, infections were the principal cause of death in our transplant patients, confirming a previous report from our center (28), and similar reports in allo-HCT recipients (33–36). Most (41%) deaths due to infections occurred in the first one-hundred days following HCT, probably related to neutropenia and poor immune function (37). No differences in IRM were observed based on HCT type, a finding also observed in other cohorts (35, 38). Other studies documented an increased IRM in haplo-HCT recipients, proposing that delayed reconstitution of the immune system after PT-Cy could lead to increased IRM (36, 39).

Cytokine release syndrome (CRS) occurs in approximately 15% of outpatient procedures compared to 70% of inpatient HCT (9). Our results are comparable to the former at a rate of 18.5% in the haplo-HCT recipients, while for the HLA-identical group it was 2.5%. CRS was more common in our haploidentical patients, which has been previously reported as a frequent complication observed in 25-90% of unmanipulated peripheral blood HCT performed in haploidentical recipients treated with PTCy for GvHD prophylaxis, although its association with increased mortality has not been demonstrated in all studies (40–43). CRS did not increase the risk for a lower OS and DFS in our cohort. Since haploidentical HCT employment is increasing, especially in LMIC, measures to prevent and treat CRS effectively should be emphasized in these settings to reduce patient morbidity. Future studies employing outpatient HCTs with RIC should report risk factors that could predict CRS occurrence and severity and help prevent its development.

The most common cause for hospitalization was neutropenic fever in both haploidentical and HLA-identical HCT, although it was more common in the former. A recent review reported that 50-80% of patients were hospitalized after an outpatient allogeneic HCT with RIC, where neutropenic fever, infections, and regimen-related complications were the most common causes (44). Hospital admission was not associated with increased mortality, yet patients with more than one hospitalization had an increased risk for lower OS and DFS. Interestingly, limited information is available comparing hospitalization rates and causes between the different transplant types. This information should be reported in additional studies due to its important association with mortality. Identifying the most frequent causes can help transplant centers using RIC to improve outcomes through better prophylactic measures, especially in LMIC, where outpatient HCT is frequently performed.

Chimerism analyses are an important tool for clinicians to predict relapse. Its use is more frequent in HTCs using non-myeloablative conditionings due to its higher risk for disease relapse (45). There is limited information on chimerism between donor types. Other studies report findings similar to ours, yet they involve patients with aplastic anemia and non-malignant diseases with different graft sources and conditionings (46–49).

It has been reported that complete chimerism (CC) at 30 days post-transplant is associated with longer relapse-free survival (50). Our study found no association with OS, DFS, NRM, and relapse in patients with CC at 30- and 100-days post-transplant. Similarly, a previous report found no association of OS and DFS with mixed chimerism (MC) at 30- and 60-days post-transplant (51). In contrast, other studies have suggested that chimerism was significantly associated with survival (52, 53). Differences between studies demonstrating significant findings were that chimerism was analyzed as a continuous variable (53), and no specific time points were described. There is no universal consensus on the ideal regimen of post-HSCT chimerism follow-up. Intervals ranging from weekly to monthly analysis have been used, and for RIC regimens, measuring chimerism at 1, 2, 3, 6, and 12 months has been proposed (54, 55). This should be further addressed as it is highly relevant for LMIC, where measuring chimerism is a significant economic burden for most patients who pay out-of-pocket for these studies and should be performed in justified time points.

Measurable residual disease (MRD) detection predicts survival and relapse in acute leukemia (54, 55). However, its association with OS, DFS, and relapse after an outpatient transplant and its pertinent time points remains inconclusive (56). Our results confirm that early positive MRD at day 30 post-HCT increases the risk for lower OS. A similar study demonstrates the clinical relevance of 30-day MRD post-transplant with survival outcomes (57). Although we did not find a higher NRM in the presence of 30-day MRD+, an increased risk for NRM in MRD+ patients has been reported (58). In our cohort, MRD positivity at day 100, but not at day 30, increased the patient’s risk for relapse at 6 months, probably reflecting the time-dependent nature of this complication. Also, the number of MRD-positive recipients at 100 days was greater than those at 30 days; thus, there were more patients at risk in the group who relapsed significantly more at 6 months. Thus, there was a correlation between MRD+ and relapse, and the discrepancies observed with the CIR in the 30-day MRD+ group will probably be solved in a larger series that include more patients at risk on day 30. These results support that MRD is more relevant for predicting relapse than chimerism, as reported in recent studies (59, 60).

It is noteworthy that no difference in most of the principal transplant characteristics, complications and outcomes, including OS, DFS, relapse, and GVHD, were found when allografted patients were compared according to type of leukemia, ALL vs. AML. This suggests that despite their known biological features and differences in clinical behavior, HSCT can give an equivalent survival probability to both types of leukemia.

In our center the graft is infused the same day it is obtained; although there were no delays in the reported cohort, it is important to consider that, due to the short viability period of hematoprogenitors, there is limited time to solve complications forcing a delay in the infusion day. These can include, for example, a receptor experiencing fever before or on day 0, or a donor not mobilizing enough cells on the transplant day; thus, a protocol to deal with events delaying or preventing the graft infusion on the scheduled day should be in place at each center.

Among the several limitations in this report, due to budgetary restrictions in the health system over the study period, partial data for MRD and chimerism are to be noted; the main reason for this is that our retrospective study spans over 10 years, and routine performance of these two laboratory measures was adopted at our center five years ago. In addition, Ph chromosome status was not available for most patients in the cohort; also, since most of our patients come from outstate and are allowed to continue their vigilance with their local hematologists after returning home to limit their out-of-pocket travel expenses, there was a relatively short follow-up. Finally, a low number of events for 30-day MRD univariate analysis could have contributed to the lack of statistical significance in Cox regression, yet this number was sufficient to yield a significant cumulative incidence for NRM.

In conclusion, outpatient HLA-identical and haploidentical transplants after reduced-intensity conditioning in adult patients with AML and ALL from a low-income population demonstrated high efficacy, achieving survival rates comparable to those reported from developed countries with inpatient procedures employing myeloablative conditioning. An acceptable safety profile was documented, although improved efforts to decrease the elevated rate of infection-related mortality are needed, which requires statistically powered collaborative efforts for defining the best strategy to deal with this potentially fatal complication.

## Data Availability

The original contributions presented in the study are included in the article/supplementary materials, further inquiries can be directed to the corresponding author/s.
